# 3-Chloro-*N*′-(4-hy­droxy­benzyl­idene)benzohydrazide

**DOI:** 10.1107/S1600536811000699

**Published:** 2011-01-12

**Authors:** Tian-Yi Li, Xiao-Li Ran

**Affiliations:** a School of Chemical Engineering, Changchun University of Technology, Changchun 130012, People’s Republic of China

## Abstract

The title compound, C_14_H_11_ClN_2_O_2_, was prepared by the reaction of 4-hy­droxy­benzaldehyde with 3-chloro­benzo­hydrazide in methanol. The dihedral angle between the two benzene rings is 38.2 (2)°. In the crystal, mol­ecules are linked through inter­molecular N—H⋯O, O—H⋯N and O—H⋯O hydrogen bonds, forming layers lying parallel to the *bc* plane.

## Related literature

For background to Schiff base compounds derived from the reaction of aldehydes with benzohydrazides, see: Pouralimardan *et al.* (2007 ▶); Dinda *et al.* (2002 ▶). For the reference bond lengths, see: Allen *et al.* (1987 ▶).
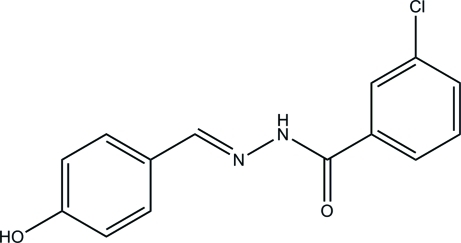


## Experimental

### 

#### Crystal data

C_14_H_11_ClN_2_O_2_
*M* *_r_* = 274.70
Orthorhombic, 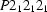

*a* = 7.547 (2) Å
*b* = 11.754 (3) Å
*c* = 14.912 (3) Å
*V* = 1322.8 (6) Å^3^
*Z* = 4
Mo *K*α radiation
μ = 0.29 mm^−1^
*T* = 298 K
0.23 × 0.20 × 0.20 mm

#### Data collection

Bruker APEXII CCD area-detector diffractometer
Absorption correction: multi-scan (*SADABS*; Bruker, 2005 ▶) *T*_min_ = 0.937, *T*_max_ = 0.945
6281 measured reflections
2834 independent reflections
1754 reflections with *I* > 2σ(*I*)
*R*_int_ = 0.051

#### Refinement

*R*[*F* ^2^ > 2σ(*F* ^2^)] = 0.050
*wR*(*F* ^2^) = 0.094
*S* = 0.95
2834 reflections
176 parameters
1 restraint
H atoms treated by a mixture of independent and constrained refinement
Δρ_max_ = 0.18 e Å^−3^
Δρ_min_ = −0.21 e Å^−3^
Absolute structure: Flack (1983 ▶), 1163 Friedel pairs
Flack parameter: −0.04 (9)

### 

Data collection: *APEX2* (Bruker, 2005 ▶); cell refinement: *SAINT* (Bruker, 2005 ▶); data reduction: *SAINT*; program(s) used to solve structure: *SHELXTL* (Sheldrick, 2008 ▶); program(s) used to refine structure: *SHELXTL*; molecular graphics: *SHELXTL*; software used to prepare material for publication: *SHELXTL*.

## Supplementary Material

Crystal structure: contains datablocks global, I. DOI: 10.1107/S1600536811000699/hg2786sup1.cif

Structure factors: contains datablocks I. DOI: 10.1107/S1600536811000699/hg2786Isup2.hkl

Additional supplementary materials:  crystallographic information; 3D view; checkCIF report

## Figures and Tables

**Table 1:** Hydrogen-bond geometry (Å, °)

*D*—H⋯*A*	*D*—H	H⋯*A*	*D*⋯*A*	*D*—H⋯*A*
N2—H2⋯O1^i^	0.90 (3)	2.14 (2)	2.996 (3)	157 (3)
O1—H1⋯N1^ii^	0.82	2.55	3.004 (3)	116
O1—H1⋯O2^ii^	0.82	1.96	2.765 (3)	168

Symmetry codes: (i) 
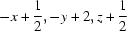
; (ii) 
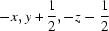
.

**Fig. 1.**
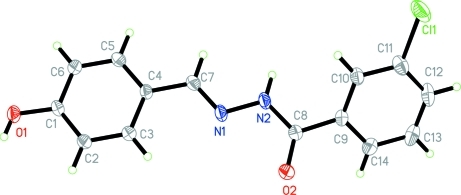
The molecular structure of the title compound with 30% probability displacement ellipsoids for non-hydrogen atoms.

**Fig. 2.**
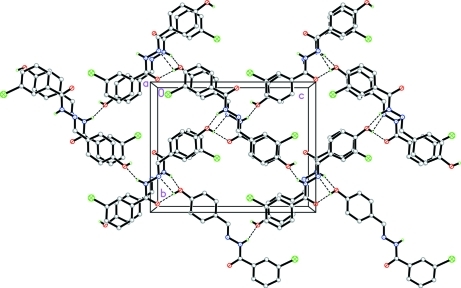
The molecular packing of the title compound. Hydrogen bonds are drawn as dashed lines.

## supplementary crystallographic information

### Comment

In the last few years, a number of Schiff bases derived from the reaction of aldehydes with benzohydrazides were prepared and structurally characterized (Pouralimardan *et al.*, 2007; Dinda *et al.*, 2002). As a continuation of the work, in the present paper, the title new Schiff base compound, Fig. 1, is reported.

The dihedral angle between the two benzene rings in the compound is 38.2 (2)°. All the bond lengths are within normal values (Allen *et al.*, 1987). The molecules are linked through intermolecular N—H···O, O—H···N, and O—H···O hydrogen bonds (Table 1) to form two-dimensional layers along the *bc* plane (Fig. 2).

### Experimental

4-Hydroxybenzaldehyde (0.122 g, 1 mmol) and 3-chlorobenzohydrazide (0.171 g, 1 mmol) were dissolved in 30 ml absolute methanol. The mixture was stirred at reflux for 10 min, and cooled to room temperature. The clear colorless solution was left to slow evaporation in air for a week, yielding colorless block-shaped crystals, which were collected by filtration and washed with methanol.

### Refinement

The amino H atom was located from a difference Fourier map and refined isotropically, with the N—H distance restrained to 0.90 (1) Å. The other H atoms were positioned geometrically and refined using the riding-model approximation, with C—H = 0.93 Å, and O—H = 0.82 Å, and *U*_iso_(H) = 1.2*U*_eq_(C) or *U*_iso_(H) = 1.5*U*_eq_(O).

### Crystal data

**Table:**

C_14_H_11_ClN_2_O_2_	*F*(000) = 568
*M**_r_* = 274.70	*D*_x_ = 1.379 Mg m^−^^3^
Orthorhombic, *P*2_1_2_1_2_1_	Mo *K*α radiation, λ = 0.71073 Å
Hall symbol: P 2ac 2ab	Cell parameters from 965 reflections
*a* = 7.547 (2) Å	θ = 2.6–25.0°
*b* = 11.754 (3) Å	µ = 0.29 mm^−^^1^
*c* = 14.912 (3) Å	*T* = 298 K
*V* = 1322.8 (6) Å^3^	Block, colorless
*Z* = 4	0.23 × 0.20 × 0.20 mm

### Data collection

**Table:**

Bruker APEXII CCD area-detector diffractometer	2834 independent reflections
Radiation source: fine-focus sealed tube	1754 reflections with *I* > 2σ(*I*)
graphite	*R*_int_ = 0.051
ω scans	θ_max_ = 27.0°, θ_min_ = 2.2°
Absorption correction: multi-scan (*SADABS*; Bruker, 2005)	*h* = −9→9
*T*_min_ = 0.937, *T*_max_ = 0.945	*k* = −12→15
6281 measured reflections	*l* = −19→16

### Refinement

**Table:**

Refinement on *F*^2^	Secondary atom site location: difference Fourier map
Least-squares matrix: full	Hydrogen site location: inferred from neighbouring sites
*R*[*F*^2^ > 2σ(*F*^2^)] = 0.050	H atoms treated by a mixture of independent and constrained refinement
*wR*(*F*^2^) = 0.094	*w* = 1/[σ^2^(*F*_o_^2^) + (0.0302*P*)^2^] where *P* = (*F*_o_^2^ + 2*F*_c_^2^)/3
*S* = 0.95	(Δ/σ)_max_ < 0.001
2834 reflections	Δρ_max_ = 0.18 e Å^−^^3^
176 parameters	Δρ_min_ = −0.21 e Å^−^^3^
1 restraint	Absolute structure: Flack (1983), 1163 Friedel pairs
Primary atom site location: structure-invariant direct methods	Flack parameter: −0.04 (9)

### Special details

**Table:**

Geometry. All e.s.d.'s (except the e.s.d. in the dihedral angle between two l.s. planes) are estimated using the full covariance matrix. The cell e.s.d.'s are taken into account individually in the estimation of e.s.d.'s in distances, angles and torsion angles; correlations between e.s.d.'s in cell parameters are only used when they are defined by crystal symmetry. An approximate (isotropic) treatment of cell e.s.d.'s is used for estimating e.s.d.'s involving l.s. planes.
Refinement. Refinement of *F*^2^ against ALL reflections. The weighted *R*-factor *wR* and goodness of fit *S* are based on *F*^2^, conventional *R*-factors *R* are based on *F*, with *F* set to zero for negative *F*^2^. The threshold expression of *F*^2^ > σ(*F*^2^) is used only for calculating *R*-factors(gt) *etc*. and is not relevant to the choice of reflections for refinement. *R*-factors based on *F*^2^ are statistically about twice as large as those based on *F*, and *R*- factors based on ALL data will be even larger.

### Fractional atomic coordinates and isotropic or equivalent isotropic displacement parameters (Å^2^)

**Table:**

	*x*	*y*	*z*	*U*_iso_*/*U*_eq_	
Cl1	0.05610 (14)	0.59347 (8)	0.39379 (5)	0.0776 (4)	
N1	0.1094 (3)	0.7672 (2)	−0.03391 (13)	0.0416 (7)	
N2	0.1281 (4)	0.7085 (2)	0.04643 (14)	0.0412 (7)	
O1	0.1358 (3)	1.14877 (15)	−0.33772 (12)	0.0440 (6)	
H1	0.0707	1.1229	−0.3767	0.066*	
O2	0.0407 (3)	0.54505 (15)	−0.02189 (11)	0.0471 (6)	
C1	0.1340 (4)	1.0781 (2)	−0.26470 (16)	0.0335 (7)	
C2	0.0762 (4)	0.9674 (2)	−0.26936 (16)	0.0363 (7)	
H2A	0.0366	0.9377	−0.3236	0.044*	
C3	0.0768 (4)	0.9002 (2)	−0.19341 (15)	0.0367 (7)	
H3	0.0373	0.8254	−0.1967	0.044*	
C4	0.1361 (4)	0.9437 (2)	−0.11182 (16)	0.0323 (7)	
C5	0.1936 (4)	1.0556 (3)	−0.10858 (19)	0.0396 (8)	
H5	0.2335	1.0855	−0.0545	0.047*	
C6	0.1928 (4)	1.1235 (3)	−0.18415 (18)	0.0388 (8)	
H6	0.2311	1.1986	−0.1811	0.047*	
C7	0.1441 (4)	0.8728 (2)	−0.03118 (16)	0.0372 (7)	
H7	0.1753	0.9059	0.0232	0.045*	
C8	0.0932 (4)	0.5954 (3)	0.04547 (16)	0.0373 (7)	
C9	0.1181 (4)	0.5358 (2)	0.13258 (17)	0.0348 (7)	
C10	0.0839 (4)	0.5875 (3)	0.21398 (16)	0.0409 (7)	
H10	0.0484	0.6632	0.2159	0.049*	
C11	0.1026 (4)	0.5266 (3)	0.29184 (18)	0.0488 (9)	
C12	0.1566 (4)	0.4149 (3)	0.2914 (2)	0.0557 (9)	
H12	0.1686	0.3751	0.3450	0.067*	
C13	0.1926 (4)	0.3627 (3)	0.2109 (2)	0.0588 (10)	
H13	0.2307	0.2875	0.2097	0.071*	
C14	0.1721 (4)	0.4225 (3)	0.1316 (2)	0.0468 (9)	
H14	0.1945	0.3866	0.0772	0.056*	
H2	0.186 (4)	0.739 (2)	0.0936 (15)	0.080*	

### Atomic displacement parameters (Å^2^)

**Table:**

	*U*^11^	*U*^22^	*U*^33^	*U*^12^	*U*^13^	*U*^23^
Cl1	0.1069 (8)	0.0920 (7)	0.0338 (4)	−0.0111 (7)	0.0067 (5)	0.0092 (4)
N1	0.059 (2)	0.0393 (16)	0.0268 (12)	−0.0055 (14)	−0.0054 (13)	0.0092 (10)
N2	0.0581 (18)	0.0387 (16)	0.0269 (13)	−0.0085 (14)	−0.0096 (13)	0.0095 (11)
O1	0.0605 (16)	0.0389 (12)	0.0327 (11)	−0.0056 (12)	−0.0066 (10)	0.0104 (9)
O2	0.0678 (15)	0.0399 (12)	0.0336 (10)	−0.0019 (12)	−0.0071 (10)	−0.0021 (9)
C1	0.0313 (16)	0.0413 (18)	0.0277 (14)	0.0012 (15)	0.0015 (12)	0.0068 (13)
C2	0.0437 (19)	0.0387 (18)	0.0265 (14)	−0.0017 (16)	−0.0044 (13)	0.0001 (12)
C3	0.0426 (19)	0.0351 (17)	0.0325 (14)	−0.0005 (16)	0.0003 (13)	0.0005 (13)
C4	0.0343 (17)	0.0340 (17)	0.0285 (14)	0.0024 (15)	−0.0012 (13)	0.0057 (12)
C5	0.049 (2)	0.042 (2)	0.0280 (15)	−0.0002 (15)	−0.0079 (13)	0.0001 (14)
C6	0.047 (2)	0.0332 (19)	0.0365 (17)	−0.0042 (15)	0.0003 (13)	0.0009 (14)
C7	0.0416 (19)	0.0413 (19)	0.0285 (15)	0.0012 (17)	−0.0040 (13)	0.0015 (13)
C8	0.041 (2)	0.0368 (18)	0.0338 (15)	−0.0008 (17)	0.0018 (13)	0.0028 (13)
C9	0.0347 (19)	0.0352 (17)	0.0344 (15)	−0.0011 (16)	−0.0020 (13)	0.0080 (12)
C10	0.0460 (19)	0.0417 (18)	0.0348 (15)	−0.0031 (16)	−0.0022 (13)	0.0090 (14)
C11	0.047 (2)	0.063 (2)	0.0371 (17)	−0.0115 (19)	−0.0014 (15)	0.0099 (15)
C12	0.052 (2)	0.062 (2)	0.054 (2)	−0.010 (2)	−0.0130 (17)	0.0299 (19)
C13	0.058 (3)	0.041 (2)	0.077 (3)	0.0030 (18)	−0.012 (2)	0.019 (2)
C14	0.049 (2)	0.039 (2)	0.052 (2)	0.0001 (17)	−0.0021 (15)	0.0042 (16)

### Geometric parameters (Å, °)

**Table:**

Cl1—C11	1.747 (3)	C5—C6	1.381 (4)
N1—C7	1.270 (3)	C5—H5	0.9300
N1—N2	1.390 (3)	C6—H6	0.9300
N2—C8	1.355 (4)	C7—H7	0.9300
N2—H2	0.90 (3)	C8—C9	1.488 (3)
O1—C1	1.370 (3)	C9—C10	1.382 (3)
O1—H1	0.8200	C9—C14	1.393 (4)
O2—C8	1.231 (3)	C10—C11	1.371 (4)
C1—C2	1.375 (4)	C10—H10	0.9300
C1—C6	1.387 (4)	C11—C12	1.375 (4)
C2—C3	1.380 (3)	C12—C13	1.376 (5)
C2—H2A	0.9300	C12—H12	0.9300
C3—C4	1.393 (3)	C13—C14	1.383 (4)
C3—H3	0.9300	C13—H13	0.9300
C4—C5	1.386 (4)	C14—H14	0.9300
C4—C7	1.464 (3)		
			
C7—N1—N2	115.9 (2)	N1—C7—H7	119.3
C8—N2—N1	117.3 (2)	C4—C7—H7	119.3
C8—N2—H2	120 (2)	O2—C8—N2	122.9 (2)
N1—N2—H2	121 (2)	O2—C8—C9	121.8 (3)
C1—O1—H1	109.5	N2—C8—C9	115.4 (2)
O1—C1—C2	122.5 (2)	C10—C9—C14	119.0 (3)
O1—C1—C6	116.9 (3)	C10—C9—C8	122.4 (3)
C2—C1—C6	120.6 (2)	C14—C9—C8	118.6 (2)
C1—C2—C3	119.9 (2)	C11—C10—C9	119.7 (3)
C1—C2—H2A	120.0	C11—C10—H10	120.1
C3—C2—H2A	120.0	C9—C10—H10	120.1
C2—C3—C4	120.5 (3)	C10—C11—C12	121.7 (3)
C2—C3—H3	119.7	C10—C11—Cl1	118.8 (3)
C4—C3—H3	119.7	C12—C11—Cl1	119.5 (2)
C5—C4—C3	118.6 (2)	C11—C12—C13	119.2 (3)
C5—C4—C7	119.9 (2)	C11—C12—H12	120.4
C3—C4—C7	121.5 (3)	C13—C12—H12	120.4
C6—C5—C4	121.2 (3)	C12—C13—C14	119.8 (3)
C6—C5—H5	119.4	C12—C13—H13	120.1
C4—C5—H5	119.4	C14—C13—H13	120.1
C5—C6—C1	119.1 (3)	C13—C14—C9	120.6 (3)
C5—C6—H6	120.5	C13—C14—H14	119.7
C1—C6—H6	120.5	C9—C14—H14	119.7
N1—C7—C4	121.4 (2)		

### Hydrogen-bond geometry (Å, °)

**Table:**

*D*—H···*A*	*D*—H	H···*A*	*D*···*A*	*D*—H···*A*
N2—H2···O1^i^	0.90 (3)	2.14 (2)	2.996 (3)	157 (3)
O1—H1···N1^ii^	0.82	2.55	3.004 (3)	116
O1—H1···O2^ii^	0.82	1.96	2.765 (3)	168

Symmetry codes: (i) −*x*+1/2, −*y*+2, *z*+1/2; (ii) −*x*, *y*+1/2, −*z*−1/2.
